# An Abnormal Condition of the Forelegs of a Colt

**Published:** 1897-10

**Authors:** H. S. Perley

**Affiliations:** St. Albans, Vt.


### AN ABNORMAL CONDITION.

By H. S. Perley, V.S.,
ST. ALBANS, VT.

The subject, a standard-bred horse colt, born March 2, 1897, was perfectly formed with the exception of his forelegs from the knees down. The metacarpal bones are twisted quarter way round, which brings the extensor tendons at the side of the leg instead of the front. There is contraction of these tendons, which bends the legs, as described, and compels the animal to walk on the side of his foot and leg as far up as the fetlock-joint.

The colt moves around without any great inconvenience, and can trot as fast as a man can run. He has been brought up on a bottle since he was three weeks old, and at the present time is growing nicely.

